# Boarding is Associated with Reduced Emergency Department Efficiency that is not Mitigated by a Provider in Triage

**DOI:** 10.5811/westjem.2020.2.45728

**Published:** 2020-04-21

**Authors:** Anthony M. Napoli, Shihab Ali, Alexis Lawrence, Janette Baird

**Affiliations:** Warren Alpert Medical School of Brown University, Department of Emergency Medicine, Providence, Rhode Island

## Abstract

**Introduction:**

Boarding of patients in the emergency department (ED) is associated with decreased ED efficiency. The provider-in-triage (PIT) model has been shown to improve ED throughput, but it is unclear how these improvements are affected by boarding. We sought to assess the effects of boarding on ED throughput and whether implementation of a PIT model mitigated those effects.

**Methods:**

We performed a multi-site retrospective review of 955 days of ED operations data at a tertiary care academic ED (AED) and a high-volume community ED (CED) before and after implementation of PIT. Key outcome variables were door to provider time (D2P), total length of stay of discharged patients (LOSD), and boarding time (admit request to ED departure [A2D]).

**Results:**

Implementation of PIT was associated with a decrease in median D2P by 22 minutes or 43% at the AED (p < 0.01), and 18 minutes (31%) at the CED (p < 0.01). LOSD also decreased by 19 minutes (5.9%) at the AED and 8 minutes (3.3%) at the CED (p<0.01). After adjusting for variations in daily census, the effect of boarding (A2D) on D2P and LOSD was unchanged, despite the implementation of PIT. At the AED, 7.7 minutes of boarding increased median D2P by one additional minute (p < 0.01), and every four minutes of boarding increased median LOSD by one minute (p < 0.01). At the CED, 7.1 minutes of boarding added one additional minute to D2P (p < 0.01), and 4.8 minutes of boarding added one minute to median LOSD (p < 0.01).

**Conclusion:**

In this retrospective, observational multicenter study, ED operational efficiency was improved with the implementation of a PIT model but worsened with boarding. The PIT model was unable to mitigate any of the effects of boarding. This suggests that PIT is associated with increased efficiency of ED intake and throughput, but boarding continues to have the same effect on ED efficiency regardless of upstream efficiency measures that may be designed to minimize its impact.

## INTRODUCTION

Emergency department (ED) visits have steadily increased over the last decade, outpacing population growth.^1^,^2^ As ED utilization has increased, boarding of admitted patients in the ED while they await inpatient bed assignment has become a nationally ubiquitous issue.^3^–^5^ In 2014, Pitts *et al.* found the national median boarding time was 79 minutes with 32% of admitted patients waiting greater than two hours for bed assignment.^6^ Among other adverse effects, boarded patients are less likely to have inpatient care initiated and boarding is associated with increased mortality.^3^,^4^,^7^

The physician-in-triage (PIT) intake model has been shown to improve ED operational efficiency in both community EDs and tertiary referral centers.^8^–^12^ Specifically, PIT is associated with improved intake (shorter door to provider times and lower left without being seen rates) and throughput (shorter lengths-of-stay), as well as other ED measures of efficiency (fewer days of ambulance diversion and shorter radiologic turnaround times).^8^–^13^

PIT models are designed to improve ED intake and throughput. The effect of boarding on availability of ED beds is one of many reasons cited for why EDs move to a PIT model. Despite this, there is little research quantifying the effect that boarding has on upstream efficiency measures with or without a PIT model in place. White *et al.* in a single-center study at a high-volume, academic, tertiary referral center found that increased boarding was associated with increased length of stay of discharged patients.^5^ However, this study was a single center study in an ED that was not staffed with a PIT operational model. The primary aim of this study was to assess the effect ED boarding has on ED intake and throughput metrics and whether implementation of a PIT model mitigated those effects. Specifically, we hypothesized the PIT model would not attenuate the effects of boarding on important ED throughput metrics, in particular door to provider time (D2P) and length of stay for discharged (LOSD) patients.

## METHODS

This was a multi-site retrospective observational cohort study of ED operations at two sites. In total, 955 days of ED operations at a tertiary care academic ED (AED) and a high-volume community ED (CED) were analyzed before and after the implementation of a PIT protocol. The institutional review boards associated with each ED approved the study.

We reported descriptive statistics on the key outcome variables using median values with interquartile ranges (IQR) and proportions calculated with 95% confidence interval (CI). Unadjusted analysis of the differences in the median values of outcome variables was conducted using Wilcoxon exact test. We conducted a series of quartile regressions, adjusting for differences in daily ED census centered at the overall ED median volume by time period (before or after PIT), to determine the effects of PIT implementation and boarding time on operational metrics as measured by the key outcome variables. These key outcome variables included the following: LOSD – the total time spent in the ED from arrival to discharge of non-admitted ED patients; D2P time – the time from arrival to provider evaluation; median active-care time – the time spent managing and dispositioning a patient, defined as LOSD less D2P; and boarding time (A2D) – the time between the admission bed request to ED departure. In these quartile regression models we examined the interaction of patient boarding time with PIT before and after its implementation to determine the effects of boarding on LOSD and D2P. Additionally, we examined the median number of patients leaving without being seen (LWBS). Statistical analyses were conducted using SAS version 9.4 (SAS Institute, Cary, NC).

## RESULTS

### Census Measures Before and After Implementation of the PIT

Across the 955 days of ED operations that data were collected, there were 250 days pre- and 705 days post-PIT implementation at the AED and 552 days pre- and 443 days post-PIT implementation at the CED. During that time, there were a total 275,981 patient visits at the AED and 190,039 visits at the CED. The median daily census at both sites significantly increased pre- to post-PIT implementation, with a median increase of 8 patients/day at the AED, a 2.8% increase in daily volume (95% CI, 0.8–4.7), and a median increase of 14 patients/day or 7.6% volume increase at the CED (95% CI, 3.8–11.4) (Table 1). The percentage of patient admissions significantly increased pre- to post-PIT implementation in both EDs (p < 0.01); however, the LWBS rate did not significantly change at either site.

**Population Health Research Capsule**

What do we already know about this issue?

Boarding of admitted patients in the emergency department (ED) is known to reduce the quality and efficiency of care of both boarded patients and other ED patients.

What was the research question?

Would a provider-in-triage (PIT) model reduce the effect that boarding has on other ED patients?

What was the major finding of the study?

Every 4–5 minutes of boarding increased the length of stay of other patients by one minute. PIT did not mitigate this effect.

How does this improve population health?

PIT improves the efficiency of ED care but does not attenuate the effect of hospital boarding. Improved hospital throughput is needed to reduce the consequences of boarding.

### Operational Metrics Before and After Implementation of the PIT

Implementation of PIT was associated with significantly shorter median LOSD at both EDs (Table 2), with a decrease of 5.9% (19 minutes) at the AED and 3.3% (8 minutes at CED). After implementation of the PIT model, we found a statistically significant decrease in D2P time at both sites, and a statistically significant increase in boarding time. D2P times demonstrated the largest shift after implementation of PIT. The median D2P time decreased by 22 minutes or 43%. The D2P was 51 minutes (IQR 37–68) pre-PIT as compared with 29 minutes post-PIT (IQR 21–41) at the AED (p < 0.01). At the CED, the D2P went from 58 minutes (IQR 38–77) to 40 (IQR 26–59), a 31% decrease (p<0.01). Post-PIT implementation median active-care time increased by 5 minutes or 1.8% at the AED (pre-PIT median time = 264 [IQR 249–282] post-PIT = 269 [IQR 251–290], p = 0.01), and 9 minutes or 5% at the CED (pre-PIT median time = 180 [IQR 170–192] post-PIT = 189 [IQR 175–205], p < 0.01). For both EDs, median A2D significantly increased (AED Δ = 13.5 minutes [95% CI: 8.1, 18.01]), p < 0.01; CED Δ = 29 minutes [95% CI: 24.2, 34.8], p < 0.01), with the increase in median A2D significantly larger at the CED (p < 0.01).

A series of quartile regressions were performed to determine the effects of PIT implementation and boarding time on operational metrics at both sites, as can be seen in Table 3. In both EDs, after adjusting for variability in overall median ED census (centered on the median patient volume relevant to pre- and post-PIT), implementation of PIT at both sites was shown to be associated with shorter median D2P (AED and CED: p < 0.01) and median LOSD (AED and CED: p < 0.01), confirming the unadjusted analyses. At the AED, the implementation of PIT was associated with a 21.1-minute reduction in D2P and a 29.8-minute reduction in LOSD, after adjusting for the effects of census and boarding. This reduction was also found in the CED with a reduction of 18.5 minutes in median D2P and 11.5 minutes in median LOSD.

### Association of Boarding with Upstream Inefficiency of ED Care

**Figure 1a–1d:**
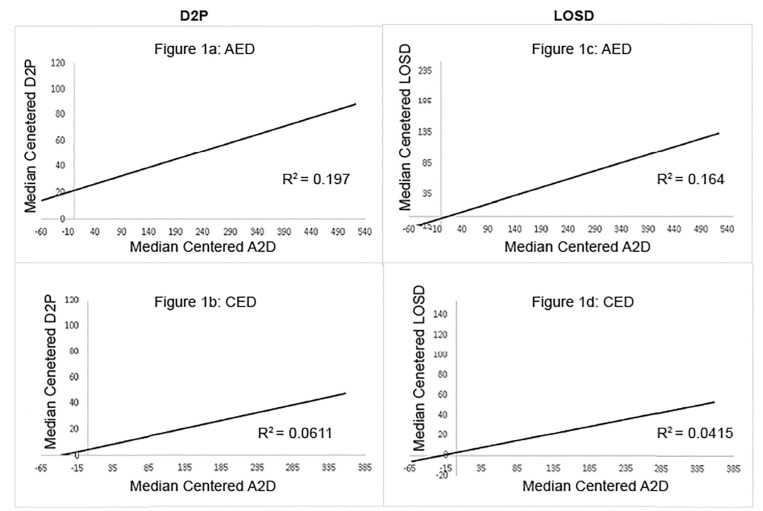
Effects of Patient Boarding on Median Centered Door to Physician and Length of Stay of Discharged Patients. In all four panels, X and Y axes in minutes. *AED*, tertiary care academic emergency department; *A2D*, admit request to departure for boarded patients awaiting hospital admission; *CED*, community emergency department; *D2P*, arrival to being seen by physician; *LOSD*, total length of stay for discharged patients;

After adjusting for median census at each site, the effect of boarding (A2D) on upstream efficiency metrics of D2P and LOSD is constant and unchanged despite the implementation of PIT. Every 7.7 minutes of boarding is associated with an additional one minute of median D2P (p < 0.01), and every four minutes of boarding is associated with an additional one minute to the median LOSD (p < 0.01) at the AED (Table 3 and Figure 1). Every 7.1 minutes of boarding is associated with an additional minute to the D2P time (p < 0.01) and every 4.8 minutes of boarding with an additional minute to the median LOSD (p < 0.01) at the CED. Even with a significant reduction in D2P and LOS after PIT implementation, with median A2D of 111 post-PIT implementation at the AED, 14.4 minutes are added to the median D2P, and 27.75 minutes are added to the median LOSD (assuming that the ED census is at the median).

The effects on the CED after PIT implementation, with a median A2D of 106 minutes, are an additional 14.84 minutes for the median patient’s D2P and 22.26 minutes on the median LOSD. Two additional analyses that we conducted (not shown in Table 2) support these results; regression slopes for A2P on D2P and LOSD were significant for both pre- and post-PIT implementation for both the AED (D2P pre-PIT p < 0.01, post p < 0.01; LOSD pre-PIT p = 0.03, post p < 0.01), and for the CED (D2P pre-PIT p = 0.02, post p < 0.01; LOSD pre-PIT p = 0<0.01, post p < 0.01). Additionally an interaction term for PIT * A2P added to the analysis shown in Table 3 for both ED sites and for the two outcomes (D2P and LOSD) were not significant (interaction A2D * PIT for AED; D2P = 0.06, LOSD = 0.17; CED D2P =0.52, LOS p = 0.44), indicating that the effect of A2P on the efficiency outcomes was not attenuated with the introduction of PIT.

## DISCUSSION

The effects of boarding have been well classified in the ED literature and national quality guidelines. The PIT model is one clear way in which ED administrative leadership has been able to improve the efficiency of intake and throughput, often in the face of worsening boarding. However, boarding continues to be a problem for EDs across the country. The output constraints of poor hospital throughput that boarding puts on EDs reduce the efficiency of ED intake and throughput.

This study showed that implementation of a PIT model was not associated with an ability to mitigate the operational inefficiencies created by boarding. PIT was associated with decreases in D2P and LOS for discharged patients at two EDs that concurrently experienced increases in ED volume and boarding. Increased boarding of admitted patients was positively associated with increased D2P and LOS for discharged patients; this relationship was unchanged despite the implementation of PIT. This suggests that increased boarding of admitted patients has systemic effects on overall ED efficiency, even affecting the care of discharged (non-boarded) patients. Improvements in ED throughput using the PIT model is largely independent of hospital throughput and could not mitigate the effects of boarding.

In 2003 Asplin *et al.* presented a conceptual framework that examined ED crowding as a combination of input, throughput, and output stressors.^14^ Input, or the demand for ED care, reflects the extent to which there is patient demand for ED services and is largely beyond the immediate control of ED leadership. Output factors, including inpatient hospital capacity and access to appropriate outpatient services are likewise outside the scope of ED administrative control. Throughput focuses on time spent within the ED and is largely comprised of active patient-care time. Interventions designed to impact throughput time include process improvements during patient intake, disease-specific protocols designed to reduce provider variability, and the implementation of clinical decision units. The PIT model is one way in which EDs can innovate to improve ED throughput efficiency. This study demonstrated the efficacy of PIT implementation on improving intake and throughput in two large, urban EDs. We were also able to quantify the negative effect that boarding has on the upstream efficiency of both EDs before and after implementation of PIT and also demonstrate that PIT was unable to mitigate this negative correlation.

Improving ED operational efficiency in the current environment of increasing ED boarding will likely require hospital-wide policy changes that address the downstream bottlenecks in care. EDs can improve operational efficiency using innovative models of care such as PIT, but outflow of patients remains a significant contributor to ED inefficiency. Viccellio et al suggested that transfer of boarding patients to inpatient hallway beds may mitigate the impact of boarding but did not directly examine the impact of a full-capacity protocol on operational metrics.^15^ Several studies have documented that there is no increased in-hospital mortality or intensive care unit transfer rate among patients in inpatient-ward hallway beds, suggesting that boarding of patients in inpatient hallway beds is not associated with increased patient harm.^15^,^16^

To the contrary, there are a number of studies that demonstrate the adverse quality of care associated with boarding patients in the ED.^4^,^17^ Surveyed patients strongly prefer waiting in inpatient wards rather than the ED.^16^,^18^ Despite these data, Pitts et al in 2014 found that only 19% of EDs used a strategy of moving admitted boarding patients to alternate sites in the hospital.^6^ One prior study was able to quantify the throughput inefficiency introduced by higher levels of boarding; however, this single-center study was at a center where a PIT model had yet to be introduced.^5^ Modern innovative models such as PIT are often a reactive process designed to improve ED throughput in the face of poor hospital throughput. However, as this study demonstrates, these models are unable to substantially mitigate the effect that boarding has on ED intake and throughput.

## LIMITATIONS

This study has several limitations. One important limitation is that these effects were quantified in daily intervals. EDs often have variable arrival and admission rates. Consequently, boarding follows a similar but often-delayed pattern.^19^,^20^ Daily data intervals likely underestimate the effects of boarding as the effects of boarding are spread over all patients. Many patients, particularly during periods of low ED census, may not be subjected to the inefficiencies introduced by boarding. Consequently, a shorter interval study would more accurately assess the effects of boarding during times of peak ED crowding.

Second, this study took place in two separate institutions under the same umbrella and leadership of one healthcare system. Thus, the system and ED leadership were largely familiar with each other’s systems of care and followed similar care pathways and surge protocols. This includes implementation of a very similar PIT model. While a similar PIT model may improve the interval validity of the study, it may limit the conclusions one may draw given the diversity of expected changes in the way healthcare systems address the issue of boarding.

Workflow changes that occurred during the implementation of the PIT model may have also confounded these results. These include expedited admission protocols, expansion of a clinical decision unit, and innovations in efficiency of core processes such as lab and radiology turnaround time. While each of these could reduce the overall LOS and overestimate the departmental efficiencies associated with the PIT model they are also likely to have a positive impact on boarding, thus underestimating our findings. Lastly, one system implemented its PIT model after learning from the operational challenges of the first.

## CONCLUSION

This study showed that implementation of a PIT model was associated with improved intake and throughput at two EDs during a time of increasing ED volume and boarding. However, the PIT model was not able to mitigate any of the upstream inefficiencies introduced by boarding. Increased boarding of admitted patients was positively associated with increased D2D and LOS for discharged patients despite the implementation of PIT. This suggests that increased boarding of admitted patients has systemic effects on overall ED efficiency affecting care of discharged (non-boarded) patients. While PIT may improve ED throughput, it cannot mitigate the negative effects of poor hospital throughput introduced by boarding.

## Figures and Tables

**Table 1:** Patient census data pre- to post-implementation of a physician in triage.

Outcome	ED type	Pre-PIT	Post-PIT	P-value
Median Daily Census (IQR)	AED	284 (271, 300)	292 (275, 306)	0.01
	CED	185 (174, 196)	199 (186, 210)	<0.01
Median daily admissions (IQR)	AED	80 (74, 87)	84 (76, 91)	<0.01
	CED	50 (45, 56)	55 (49, 61)	<0.01
Mean annual percent admit (SD)	AED	28.2 (±2.8)	29.3 (±2.8)	<0.01
	CED	26.7 % (±3.5)	27.6 % (± 3.7)	<0.01
Median daily LWBS (IQR)	AED	11 (6, 19)	11 (6, 17)	0.13
	CED	5 (2, 8)	4 (2, 9)	0.29
Mean annual percent LWBS (SD)	AED	4.6 % (± 2.3)	4.1 % (± 2.3)	0.15
	CED	3.2 % (±1.3)	2.9 % (± 1.2)	0.24

*ED*, emergency department; *AED*, tertiary care academic emergency department; *CED*, community emergency department; *LWBS*, left without being seen; *PIT*, physician in triage; *IQR*, interquartile range; *SD*, standard deviation.

**Table 2:** Operational metrics pre- to post-implementation of a physician in triage.

Metric (min)	ED type	Pre-PIT Median (IQR)	Post-PIT Median (IQR)	P-value
Discharged patients				

D2P	AED	51 (37, 68)	29 (21, 41)	<0.01
	CED	58 (38, 77)	40 (26, 59)	<0.01
LOSD	AED	289 (257, 320)	261 (238, 297)	<0.01
	CED	241 (219, 264)	232.5 (209, 265)	0.01

Admitted patients				

A2D	AED	97.5 (84.5, 116)	111.0 (93, 144.5)	<0.01
	CED	77 (66,92)	106 (80,140)	<0.01

*ED*, emergency department; *AED*, tertiary care academic emergency department; *CED*, community emergency department; *D2P*, arrival to being seen by physician; *LOSD*, total length of stay for discharged patients; *A2D*, admit request to departure for boarded patients awaiting hospital admission; *PIT*, physician in triage.

**Table 3:** Quartile regression models examining the effect of boarding on median door-to-provider time and median discharged patient length of stay (in minutes).

Parameter	AED			CED		
	
	Estimate Minutes (95%CI)	SE	t-value, p-value	Estimate Minutes (95%CI)	SE	t-value, p-value
D2P						

Intercept	36.84 (33.18, 40.50)	1.87	19.74, < 0.01	45.11 (42.1, 50.19)	2.08	22.18, < 0.01
Census median centered	0.32 (0.28, 0.36)	0.02	15.62, < 0.01	0.82 (0.73, 0.82)	0.04	19.88, < 0.01
A2D	0.13 (0.10, 0.16)	0.02	8.08, < 0.01	0.14 (0.10, 0.18)	0.02	6.65, < 0.01
PIT (post vs pre)	−21.61 (−23.78, −19.44)	1.11	19.44, < 0.01	−18.45 (−21.37, −15.52)	1.50	12.36, < 0.01

LOSD						

Intercept	260.7 (251.27, 270.13)	4.81	54.23, < 0.01	223.47 (216.42, 230.52)	3.59	62.2, < 0.01
Census median centered	0.66 (0.53, 0.78)	0.06	10.67, < 0.01	0.90 (0.80, 1.01)	0.05	16.91, < 0.01
A2D	0.25 (0.19, 0.31)	0.03	8.52, < 0.01	0.21 (0.13, 0.28)	0.04	5.08, < 0.01
PIT (post vs pre)	−29.83 (−38.03, −21.68)	4.17	7.16, < 0.01	−11.45 (−16.16, −4.77)	2.40	4.77, < 0.01

*ED*, emergency department; *AED*, tertiary care academic emergency department; *CED*, community emergency department; *D2P*, arrival to being seen by physician; *LOSD*, total length of stay for discharged patients; *A2D*, admit request to departure for boarded patients awaiting hospital admission; *PIT*, physician in triage; *95% CI*, 95% confidence interval; *SE*, standard error.

## References

[1] Pitts S, Pines JM, Handrigan MT (2012). National trends in emergency department occupancy, 2001 to 2008: effect of inpatient admissions versus emergency department practice intensity. Ann Emerg Med.

[2] Tang N, Stein J, Hsia RY (2010). Trends and characteristics of US emergency department visits, 1997–2007. JAMA.

[3] Coil CJ, Flood JD, Belyeu BM (2016). The effect of emergency department boarding on order completion. Ann Emerg Med.

[4] Singer AJ, Thode HC, Viccellio P (2011). The association between length of emergency department boarding and mortality. Acad Emerg Med.

[5] White BA, Biddinger PD, Chang Y (2013). Boarding inpatients in the emergency department increases discharged patient length of stay. J Emerg Med.

[6] Pitts SR, Vaughns FL, Gautreau MA (2014). A cross-sectional study of emergency department boarding practices in the United States. Acad Emerg Med.

[7] Sprivulis PC, Da Silva JA, Jacobs IG (2006). The association between hospital overcrowding and mortality among patients admitted via Western Australian emergency departments. Med J Aust.

[8] Holroyd BR, Bullard MJ, Latoszek K (2007). Impact of a triage liaison physician on emergency department overcrowding and throughput: a randomized controlled trial. Acad Emerg Med.

[9] Imperato J, Morris DS, Binder D (2012). Physician in triage improves emergency department patient throughput. Intern Emerg Med.

[10] Choi YF, Wong TW, Lau CC (2006). Triage rapid initial assessment by doctor (TRIAD) improves waiting time and processing time of the emergency department. Emerg Med J.

[11] Partovi SN, Nelson BK, Bryan ED (2001). Faculty triage shortens emergency department length of stay. Acad Emerg Med.

[12] Subash F, Dunn F, McNicholl B (2004). Team triage improves emergency department efficiency. Emerg Med J.

[13] Rowe BH, Guo X, Villa–zRoel C (2011). The role of triage liaison physicians on mitigating overcrowding in emergency departments: a systematic review. Acad Emerg Med.

[14] Asplin BR, Magid DJ, Rhodes KV (2003). A conceptual model of emergency department crowding. Ann Emerg Med.

[15] Viccellio A, Santora C, Singer AJ (2009). The association between transfer of emergency department boarders to inpatient hallways and mortality: a 4-year experience. Ann Emerg Med.

[16] Viccellio P, Zito JA, Sayage V (2013). Patients overwhelmingly prefer inpatient boarding to emergency department boarding. J Emerg Med.

[17] Carr BG, Hollander JE, Baxt WG (2010). Trends in boarding of admitted patients in US emergency departments 2003–2005. J Emerg Med.

[18] McGowan H, Gopeesingh K, O’Kelly P (2018). Emergency department overcrowding and the full capacity protocol cross over study: what patients who have experienced both think about being an extra patient in the emergency department or on a ward. Ir Med J.

[19] Gorski JK, Batt RJ, Otles E (2017). The Impact of emergency department census on the decision to admit. Acad Emerg Med.

[20] McCarthy ML, Aronsky D, Jones ID (2008). The emergency department occupancy rate: a simple measure of emergency department crowding?. Ann Emerg Med.

